# Effect of intensive water-salt diet nursing intervention on blood pressure and volume load in patients with chronic renal failure

**DOI:** 10.1080/0886022X.2025.2474854

**Published:** 2025-03-11

**Authors:** Liyan Wu, Wanli Ma, Hui Zhang, Ting Yang, Mengxi Sun, Zhen Yang, Xiaohan Guo

**Affiliations:** Hemodialysis Center, Affiliated Hospital of Shandong University of traditional Chinese medicine, Jinan, China

**Keywords:** Chronic renal failure, water-salt diet, nursing intervention, blood pressure, volume load

## Abstract

**Background:**

This study aimed to investigate the impact of a comprehensive nursing intervention targeting high water and salt intake on blood pressure and volume burden in patients with chronic renal failure.

**Method:**

From January 2020 to January 2023, 120 patients diagnosed with chronic renal failure were treated at our hospital. Participants were randomly allocated to either a control group (*n* = 60) receiving standard dietary education or an observation group (*n* = 60) receiving intensive water-salt diet nursing intervention alongside standard education. Blood pressure, volume load, and related parameters were compared after a 6-month observation period.

**Result:**

Both groups exhibited reduced systolic and diastolic blood pressure post-intervention (*p* < 0.05). The observation group demonstrated a significantly lower extracellular water-to-total body water ratio (ECW/TBW) compared to the control group (*p* < 0.05). The observation group also showed higher 24-hour urine volume (*p* < 0.05), hemoglobin levels, creatinine clearance rates (*p* < 0.05), and treatment compliance (*p* < 0.05), alongside a lower complication rate (3.33% vs. 13.33%; χ^2^ = 3.927, *p* < 0.05). A negative correlation was observed between the Therapeutic Intervention Scoring System (TISS) scale and post-intervention blood pressure/volume load (r = −2.924, −2.184; *p* < 0.05).

**Conclusion:**

Intensive water-salt diet nursing interventions effectively control blood pressure, reduce volume load, and mitigate complications in chronic renal failure patients. This approach should be widely implemented in clinical practice.

## Introduction

Chronic renal failure (CRF) is primarily caused by progressive renal parenchymal damage due to various etiologies, leading to renal atrophy, dysregulated water-electrolyte balance, metabolic waste retention, and multi-system complications [1]. Maintenance hemodialysis remains the cornerstone treatment for CRF, effectively prolonging survival and reducing mortality. However, long-term hemodialysis may result in inadequate elimination of metabolic waste and fluid excess, contributing to volume overload [2]. This condition is closely associated with hypertension and heart failure. Rapid ultrafiltration during dialysis increases the risk of acute complications such as hypotension and muscle cramps [3]. Consequently, adherence to low-sodium or sodium-restricted diets is critical for improving prognosis and quality of life. Current guidelines for daily sodium intake in dialysis patients vary internationally, ranging from 3.0 to 6.0 g/d [4]. Notably, compliance with dialysis regimens, dietary restrictions, and fluid control tends to decline with prolonged treatment duration [5], underscoring the necessity for rigorous water and salt management in CRF patients [6]. To evaluate the impact of intensive water and salt dietary interventions on blood pressure and volume load, we conducted a 6-month observational study involving 120 CRF patients undergoing maintenance hemodialysis.

## Materials and methods

### Study population

This study involved 120 patients diagnosed with chronic renal failure who received treatment at our hospital between January 2020 and January 2023. Prior to participation, all patients were provided with detailed information about the study and voluntarily signed an informed consent form. The research protocol was reviewed and approved by the hospital ethics committee. Participants were randomly assigned to either the observation group (*n* = 60) or the control group (*n* = 60). Nursing interventions and subsequent follow-up were conducted over a six-month period.

### Inclusion and exclusion criteria

The inclusion criteria for this study required participants to meet the diagnostic criteria for chronic renal failure and to have normal cognitive function with the ability to communicate effectively. Participants were excluded if they had a cardiac pacemaker, cardiovascular or infectious diseases, or neurological disorders.

### Control group intervention

All patients in this study received routine dialysis treatment throughout the intervention period. In the control group, patients were provided with standard dietary education aimed at maintaining renal function. The focus was on guiding patients to adjust their diet based on kidney function, with particular attention to controlling protein, salt, potassium, and phosphorus intake, as well as maintaining water balance. The goal was to reduce the burden on the kidneys through an appropriate diet and to slow the progression of chronic renal failure. Education was delivered primarily through oral courses and the distribution of written materials, which provided patients with basic knowledge on nutrition and renal care.

### Observation group intervention

In contrast, the observation group received an enhanced, more personalized approach to dietary management. This involved in-depth explanations of the importance of restricting water and salt intake, targeting both patients and their families. The objective was to deepen the patients’ understanding and equip them with the practical skills necessary to manage their intake effectively. Additionally, family members and caregivers were encouraged to actively participate in the education process, thus contributing to a comprehensive support network for the patients. Educational content was tailored to each patient’s individual circumstances, taking into account factors such as age, condition severity, symptoms, educational background, and cultural beliefs. This approach ensured that the intervention was relevant and effective for each patient.

### Multichannel health education

Health education in the observation group was continuous and delivered through multiple channels such as face-to-face communication, WeChat, telephone consultations, and a variety of online resources (including websites, books, and television programs). This multi-channel approach ensured that patients received consistent and comprehensive information. The medical team also guided patients in developing the habit of regular self-weighing and tracking daily food, water, and urine intake to ensure accurate monitoring of their dietary habits. To reinforce the intervention, weekly follow-up visits were conducted by designated nurses who closely monitored dietary intake, provided additional educational support, and assessed treatment compliance. During these follow-up visits, the diet management team evaluated patient adherence to the dietary regimen and adjusted the intervention plan as necessary based on the individual’s progress and challenges.

### Illustrative case studies and food diary method

To make the education more tangible, illustrative case studies were used, demonstrating the adverse effects of excessive water intake and high-sodium diets on cardiovascular health and weight gain during dialysis. These case studies helped patients intuitively understand the significance of controlling their water and sodium intake. Patients were also asked to maintain a detailed food diary, recording all consumed foods and beverages, including types, portions, and times. This method helped ensure that dietary data was accurately tracked and that patients could self-monitor their intake more effectively.

### Dietary compliance assessment

For evaluating dietary compliance, a comprehensive approach was used, taking into account the patient’s food diary, weight changes, urine volume records, and the nurse’s observations during follow-up visits. This data was then analyzed to identify potential problems, allowing for early intervention if necessary. The goal was to ensure that each patient adhered to the dietary recommendations, as this was essential for managing their chronic renal failure effectively.

### Measurement of dietary salt intake and water intake

Regarding dietary salt intake, urine samples were collected at 8:10 AM from each patient. Urine sodium and potassium levels were measured using ion-selective methods, while creatinine was assessed using the dynamic enzymatic hydrolysis method. These measurements allowed for the calculation of 24-h urinary sodium and potassium excretion. The daily salt intake was estimated based on the proportion of urine sodium excretion (1 g of urine sodium corresponds to approximately 2.55 g of dietary salt intake). Additionally, the water content in foods and beverages was calculated using the China Food Composition Table (2016), which provided an accurate assessment of total daily water intake.

### Echocardiographic assessment

Echocardiography was performed for all patients both at the time of enrollment and after the intervention to assess changes in cardiac function, specifically left ventricular end-diastolic diameter (LVIDd) and ejection fraction (EF). These measurements were crucial for evaluating the impact of the dietary intervention on cardiovascular health in patients undergoing dialysis.

### Compliance with treatment measures

Compliance with treatment measures was assessed using a questionnaire designed to evaluate the implementation of nursing interventions, including diet, physical activity, and medical treatment. The questionnaire covered 10 key aspects, such as medication adherence, pain management, psychological support, and the management of complications. The nurse in charge observed and recorded patient cooperation throughout the treatment process. Patients who actively cooperated with the medical staff were classified as having excellent compliance, those who followed the instructions after several explanations were considered to have good compliance, and those with difficulty adhering to treatment plans were categorized as having poor compliance.

### Evaluation of nursing intervention effectiveness

The effectiveness of the nursing intervention was evaluated using the Therapeutic Intervention Scoring System (TISS-28), a simplified version of the TISS-76 developed by Miranda in 1996. The TISS-28 includes seven assessment categories: basic treatment, respiratory support, cardiovascular support, renal support, nervous system support, metabolic support, and special interventions. Each item is scored on a scale of 1 to 8, with the total score reflecting the overall effectiveness of the nursing interventions.

### Observation parameters

Following the implementation of the dietary intervention, several key parameters were assessed for changes: (1) blood pressure, (2) edema index (ECW/TBW), (3) left ventricular end-diastolic diameter (LVIDd) and cardiac ejection fraction (EF), and (4) average 24-h urine volume. These measurements were recorded at both the baseline and after six months of intervention.

### Statistical analysis

Data were analyzed using SPSS 25.0 software (IBM, Armonk, NY, USA). Continuous variables that followed a normal distribution were presented as mean ± standard deviation (x̅ ± s). Comparisons between groups were performed using the t-test, while categorical data were expressed as percentages (%) and analyzed using the χ^2^ test. A p-value of less than 0.05 was considered statistically significant, and all tests were performed using a two-tailed approach.

## Results

### Comparison of basic data between the two groups

A comparison of the basic data between the two groups revealed no significant differences (*p* > 0.05). Table 1 shows the comparability of the two groups. After the intervention, the water and salt intake of patients in the observation group was significantly lower than that of the control group (*p* < 0.05).

**Table 1.** Comparison of basic data of two groups of subjects.

Project.	Observation group.	Control group	*t*/*χ*^2^	*p*
	(*n* = 60)	(*n* = 60)		
Age	60.62 ± 4.71	61.12 ± 5.13	0.262	0.774
Sex (n%)				
Male	42 (70.00%)	39 (65.00%)	1.117	0.239
Female	18 (30.00%)	21 (35.00%)		
BMI (kg/m^2^)	23.96 ± 3.18	23.76 ± 4.91	1.447	0.197
Course of disease	7.52 ± 2.09	7.39 ± 2.46	0.157	0.833
Blood pressure (mmHg)				
Systolic blood pressure	166.71 ± 7.24	164.49 ± 8.62	0.674	0.529
Diastolic pressure	114.72 ± 4.33	113.66 ± 5.29	0.776	0.507
ECW/TBW	0.41 ± 0.01	0.40 ± 0.08	0.116	0.961
LVIDd	52.28 ± 4.67	51.22 ± 6.39	0.367	0.714
EF	62.47 ± 8.13	62.78 ± 5.17	0.627	0.571
Average 24-hour urine output	1,106.67 ± 441.33	1,097.61 ± 479.62	0.669	0.506
LAVi (mL/m^2^)	32.51 ± 6.31	31.57 ± 6.20	0.823	0.412
E/A	1.08 ± 0.05	1.07 ± 0.03	1.328	0.187
Use of prophylactic antibacterial drugs (n%)				
NO	34 (56.67%)	36 (60.00%)	0.137	0.711
YES	26 (43.33%)	24 (40.00%)		
Use of antihypertensive drugs				
NO	37 (61.67%)	35 (58.33%)	0.139	0.709
YES	23 (38.33%)	25 (41.67%)		
after intervention dietary salt intake (g/d)				
<6.93	32 (53.33%)	19 (31.67%)	8.066	0.045
6.93 ∼ 8.23	19 (31.67%)	23 (38.33%)		
8.24 ∼ 9.43	8 (13.33%)	12 (20.00%)		
≥9.43	1 (1.67%)	6 (10.00%)		
after intervention water intake (ml)	2,012.81 ± 293.45	2,190.94 ± 243.54	3.618	0.000

### Comparison of blood pressure levels before and 6 months after dietary intervention

As shown in Table 2, after six months of dietary intervention, both systolic and diastolic blood pressure decreased in both groups compared to baseline levels. However, the reduction was more pronounced in the observation group, with a statistically significant difference compared to the control group (*p* < 0.05).

**Table 2.** Comparison of blood pressure levels between the two groups before and 6 months after dietary intervention (X ± S).

Group.	Systolic blood pressure before intervention.	Systolic blood pressure after intervention.	Diastolic pressure before intervention.	Diastolic pressure after intervention
Observation group	166.71 ± 7.24	142.83 ± 6.37**	114.72 ± 4.33	96.76 ± 5.61**
Control group	164.49 ± 8.62	156.36 ± 7.16**	113.66 ± 5.29	107.29 ± 4.97**
*t*	0.674	3.854	0.776	2.764
*p*	0.529	<0.001	0.507	0.003

Note

* means *p* < 0.05 compared with before intervention,

** means *p* < 0.001 compared with before intervention.

### Comparison of ECW/TBW before and after dietary intervention

Following the intervention, the extracellular water-to-total body water ratio (ECW/TBW) decreased significantly in the observation group compared to baseline (*p* < 0.05). In contrast, there was no significant change in ECW/TBW in the control group after the intervention (*p* > 0.05). While there was no statistical difference in ECW/TBW between the two groups before the intervention, the observation group had a significantly lower ratio than the control group after the intervention (*p* < 0.05) (Table 3).

**Table 3.** Comparison of ECW/TBW between the two groups before and 6 months after dietary intervention (X ± S).

Group.	ECW/TBW before intervention	ECW/TBW after intervention	*t*	*p*
Observation group	0.41 ± 0.01	0.39 ± 0.02	1.042	0.031
Control group	0.40 ± 0.08	0.40 ± 0.01	0.114	0.971
*t*	0.961	3.464	–	–
*p*	0.339	0.001	–	–

### Comparison of left ventricular end-diastolic diameter (LVIDd) and cardiac ejection fraction (EF) before and after dietary intervention

After six months of dietary intervention, the mean LVIDd in the observation group decreased, accompanied by an increase in EF. However, no significant difference was observed between the two groups. In the control group, LVIDd and EF increased post-intervention, but no statistically significant differences were detected (Table 4).

**Table 4.** Comparison of LVIDd and EF between the two groups before and 6 months after dietary intervention (X ± S).

Group.	LVIDd before intervention.	LVIDd after intervention.	EF before intervention.	EF after intervention
Observation group	52.28 ± 4.67	51.23 ± 5.16	62.47 ± 8.13	63.78 ± 8.02
Control group	51.22 ± 6.39	51.47 ± 5.96	62.78 ± 5.17	63.26 ± 4.61
*t*	0.367	0.185	0.627	0.681
*p*	0.714	0.847	0.571	0.496

Note

* means *p* < 0.05 compared with before intervention,

** means *p* < 0.001 compared with before intervention.

### Comparison of average 24-hour urine volume before and after dietary intervention

Table 5 shows that the average 24-h urine volume decreased in both groups after the intervention compared to baseline levels. However, the observation group still had a significantly higher urine volume than the control group (*p* < 0.05).

**Table 5.** Comparison of average 24-h urine volume between the two groups before and 6 months after dietary intervention (X ± S).

Group.	Number of cases.	Before intervention.	After intervention	*t*	*p*
Observation group	60	1,106.67 ± 441.33	784.27 ± 440.16	4.007	<0.001
Control group	60	1,097.61 ± 479.62	455.82 ± 387.91	8.059	<0.001
*t*	/	0.669	3.851		–
*p*	/	0.506	<0.001		–

### Changes in creatinine clearance rate and hemoglobin levels before and after intervention

As shown in Table 6, the hemoglobin and creatinine clearance rates in the observation group were significantly higher than those in the control group after the intervention (*p* < 0.05).

**Table 6.** Changes in the levels of creatinine clearance rate and hemoglobulin levels before and after intervention (X ± S).

Group.	Before intervention creatinine clearance rate (ml/min)	After intervention creatinine clearance rate (ml/min)	Before intervention hemoglobin levels (g/L)	After intervention hemoglobin levels (g/L)
Observation group	3.92 ± 1.02	4.71 ± 1.15	88.43 ± 11.82	97.82 ± 12.85
Control group	3.97 ± 1.05	4.05 ± 1.08	88.37 ± 12.05	91.05 ± 11.58
*t*				
*p*				

### Comparison of treatment compliance between the two groups

Table 7 indicates that treatment compliance was significantly higher in the observation group than in the control group (*p* < 0.05).

**Table 7.** Comparison of treatment compliance between two groups of patients.

Group.	Number of cases.	Excellent	Good	Bad	Excellent and good rate
Observation group	60	51	8	1	98.33
Control group	60	34	15	11	81.67
*χ* ^2^	/				9.259
*p*	/				0.002

### Changes in antihypertensive drug use before and after intervention

Table 8 shows that, although there was no statistically significant difference in the overall use of antihypertensive medications between the two groups, a higher number of patients in the observation group switched to safer diuretics and calcium antagonists after the intervention.

**Table 8.** Changes in intensity of antihypertensive drug use before and after intervention.

Group.	Number of cases.	Before intervention				
		Beta-blockers	diuretics	Alpha-blockers	Calcium antagonists	Angiotensin inhibitors
Observation group	26	4	5	6	6	5
Control group	24	4	4	6	5	4
*χ* ^2^	/	0.015	0.056	0.025	0.037	0.056
*p*	/	0.902	0.814	0.874	0.848	0.814

**Table:** 

After intervention				
Beta blockers	diuretics	alpha blockers	calcium antagonists	Angiotensin inhibitors
2	10	2	9	3
3	5	6	5	4
0.321	1.847	2.782	1.176	0.273
0.571	0.174	0.095	0.278	0.602

### Correlation analysis

Pearson’s linear correlation analysis showed that the TISS score was negatively correlated with post-intervention blood pressure and post-intervention volume load (r = −2.924, −2.184; *p* < 0.05).

### Comparison of complication rates

The complication rate in the observation group was significantly lower (3.33%) compared to the control group (13.33%) (χ^2^ = 3.927, *p* < 0.05).

## Discussion

Patients who have chronic renal failure are susceptible to hypertension and volume overload due to the decline in renal metabolic function, hindered fluid excretion, and imbalances in water and salt metabolism. This leads to an excessive cardiac preload and afterload [7]. Initially, the myocardium compensates for these changes by adaptively altering the length of muscle fibers and increasing contractility. However, over time, this can cause irreversible damage to cardiac function [8]. Sodium salt is predominantly obtained from food, with a near 100% absorption rate. Sodium ions are the primary electrolytes in the extracellular fluid and regulate water balance, pH, and osmotic pressure. Since the kidneys excrete approximately 95% of sodium ions *via* urine, any imbalance in sodium processing due to chronic renal failure can lead to increased extracellular fluid osmotic pressure, excessive thirst, water retention, and subsequently, hypertension, heart failure, and pulmonary edema [9,10]. In patients with chronic renal failure, the excessive ultrafiltration volume and rapid ultrafiltration rate during dialysis lead to a rapid decrease in volume load, making it challenging to maintain equilibrium among intracellular fluid, extracellular fluid, and capillary volume. This can result in various acute complications such as hypotension, muscle spasms, arrhythmia, angina pectoris, and in severe cases, even shock, posing a threat to the patient’s life and safety [11]. Additionally, it can further deteriorate residual renal function and increase the risk of cardiovascular events. Research has demonstrated that strict control of dietary sodium intake among dialysis patients can effectively reduce serum sodium concentration and the incidence of volume overload [12]. Therefore, reducing water and sodium retention primarily revolves around managing sodium consumption. The therapeutic outcomes for patients with chronic renal failure significantly rely on treatment compliance, particularly maintaining a low-sodium diet, as noncompliance is an independent risk factor for various complications, resulting in increased mortality rates [13]. Recent studies have revealed a direct association between sodium intake and mortality in patients with chronic renal failure [14]. Thus, emphasizing dietary counseling and education to promote water and salt intake management is of utmost importance in controlling the disease.

This study aimed to investigate the effects of a diet intervention on patients with chronic renal failure over a period of 6 months. The control group received customary diet guidance, while the observation group received intensive guidance specifically targeting water and salt intake, in addition to the customary guidance. This intensified guidance aimed to improve the understanding and compliance of the patients in managing their water and salt intake. The management of water and salt intake is a continuous process that should be integrated into the patients’ daily lives. To ensure this, the responsible nurses conducted weekly follow-ups and evaluations of the patients’ eating habits and weight gain during dialysis. They supervised their eating behaviors, fine-tuned and quantified the process. Moreover, the patients were encouraged to actively participate in discussions and ask questions about their diet. The medical staff patiently listened to the patients’ efforts in controlling their diet, motivating their self-initiative in salt control, enhancing their self-confidence and ability to self-manage, and gradually modifying their unhealthy eating habits. The results of this study revealed that the observation group exhibited better blood pressure control compared to the control group after 6 months of dietary intervention (*p* < 0.05). Additionally, both groups experienced a decrease in average 24-h urine volume after the intervention, with the observation group showing a higher decrease than the control group (*p* < 0.001). These findings indicate that strengthening the management of water and salt intake can effectively reduce dialysis-induced weight gain in patients with chronic renal failure, regulate the reduction in urine volume caused by rapid ultrafiltration, and promote the preservation of residual renal function. Numerous studies have demonstrated that residual renal function is closely associated with the survival and mortality rates of dialysis patients. The decline in residual renal function is directly proportional to the occurrence of blood volume and cardiovascular diseases [15–17].

Even a minimal level of residual kidney function can decrease the mortality rate of individuals with chronic renal failure, effectively enhance the blood phosphorus and anemia levels in dialysis patients with chronic renal failure, and decrease the occurrence and severity of sleep disorders and skin itching [18]. It is closely associated with the patients’ blood pressure levels. Therefore, protecting the residual kidney function in hemodialysis patients should be prioritized. Excess fluid volume is not only a common complication among hemodialysis patients, but also a significant contributor to cardiovascular and cerebrovascular incidents in this population. An interdialysis volume increase of over 5% is linked to a 40% rise in cardio-cerebrovascular events [19]. Prolonged excess fluid volume is an independent risk factor for mortality in hemodialysis patients. The ratio of extracellular water (ECW) to total body water (TBW), known as the edema index, exhibits strong consistency and can serve as a reference standard for evaluating patients’ fluid volume load [20]. Some studies even propose that the ECW/TBW ratio can be regarded as a crucial indicator for evaluating capacity load [21]. The findings of this investigation revealed that, after intensive water and salt diet nursing, the ECW/TBW of patients in the observation group significantly decreased, whereas there were no significant differences in the ECW/TBW of the control group following intervention. These results demonstrate the effectiveness of intensive water and salt diet nursing in reducing fluid volume load in individuals with chronic renal failure. Additionally, the study showed that after 6 months of dietary intervention, the average LVIDd value in the observation group was lower and the EF was higher compared to before treatment, whereas there were no significant changes in LVIDd and EF in the control group post-treatment, indicating an inability to effectively depict changes in fluid volume load before and after treatment in chronic renal failure patients. This outcome may be attributed to the prolonged disease duration and irreparable damage to cardiomyocytes, further emphasizing the importance of early intensive water and salt diet nursing for chronic renal failure patients.

Although this study reveals the positive impact of intensive water-salt diet nursing intervention on blood pressure and volume load in patients with chronic renal failure, there are still some deficiencies that need to be improved in future research. First, the sample size selected for this study was relatively small, with only 120 patients, all from the same hospital, which may limit the representativeness and universality of the results. In order to more accurately assess the effect of intervention, future studies should consider expanding the sample size and including patients from different regions and hospitals. Secondly, the observation period is only 6 months, which may not be enough to comprehensively assess the impact of long-term intervention on the patient’s condition. Chronic renal failure is a long-term chronic disease, and its disease development and treatment effect often require longer observation. Therefore, future research should extend the observation period to gain a deeper understanding of the long-term effects of intensive water-salt diet nursing intervention. In addition, this study mainly focused on changes in physiological indicators such as blood pressure and volume load, but lacked evaluation of patients ‘quality of life and mental state. These aspects are equally important for a comprehensive evaluation of treatment effects. Therefore, future research should comprehensively consider multiple dimensions to more comprehensively assess the impact of intensified water-salt diet nursing intervention on patients with chronic renal failure.

In conclusion, the implementation of a comprehensive water and salt restriction regimen for patients with chronic renal failure facilitates their gradual adaptation to and conscious acceptance of dietary limitations. This, in turn, improves overall dietary compliance. Additionally, such a regimen plays a critical role in enhancing the management of blood pressure and volume load in these patients. Therefore, it is essential that this nursing practice be widely promoted and integrated into clinical settings, as it effectively prevents or reduces water and sodium retention and mitigates associated complications in patients with chronic renal failure.

## References

[1] Carney EF. The impact of chronic kidney disease on global health. Nat Rev Nephrol. 2020;16(5):251–251. doi:10.1038/s41581-020-0268-7. 32144399

[2] Ke C, Liang J, Liu M, et al. Burden of chronic kidney disease and its risk-attributable burden in 137 low-and middle-income countries, 1990-2019: results from the global burden of disease study 2019. BMC Nephrol. 2022;23(1):17. doi:10.1186/s12882-021-02597-3. 34986789 PMC8727977

[3] Bellizzi V, Signoriello S, Minutolo R, et al. No additional benefit of prescribing a very low-protein diet in patients with advanced chronic kidney disease under regular nephrology care: a pragmatic, randomized, controlled trial. Am J Clin Nutr. 2022;115(5):1404–1417. doi:10.1093/ajcn/nqab417. 34967847

[4] Praditpornsilpa K, Garneata L, Lin YC, et al. Economic analysis of a ketoanalogue-supplemented very low-protein diet in patients with chronic kidney disease in Taiwan and Thailand. J Ren Nutr. 2023;33(2):269–277. doi:10.1053/j.jrn.2022.09.004. 36179957

[5] Milazi M, Bonner A, Douglas C. Effectiveness of educational or behavioral interventions on adherence to phosphate control in adults receiving hemodialysis: a systematic review. JBI Database System Rev Implement Rep. 2017;15(4):971–1010. doi:10.11124/JBISRIR-2017-003360. 28398983

[6] Milazi M, Douglas C, Bonner A. A bundled phosphate control intervention (4Ds) for adults with end-stage kidney disease receiving haemodialysis: a cluster randomized controlled trial. J Adv Nurs. 2021;77(3):1345–1356. doi:10.1111/jan.14700. 33277736

[7] Zhao X, Dong Q, Zhao G, et al. Effects of an Omaha system-based continuing nursing program on nutritional status in patients undergoing peritoneal dialysis: a randomized controlled trial. Int Urol Nephrol. 2020;52(5):981–989. doi:10.1007/s11255-020-02449-3. 32232721

[8] You L, Wang X, Wang W. A novel substrate-inspired fluorescence-based albumin detection improves assessment of clinical outcomes in hemodialysis patients receiving a nursing nutrition intervention. Med Sci Monit. 2021;27:e930257. doi:10.12659/MSM.930257. 34375323 PMC8364288

[9] Arad M, Goli R, Parizad N, et al. Do the patient education program and nurse-led telephone follow-up improve treatment adherence in hemodialysis patients? A randomized controlled trial. BMC Nephrol. 2021;22(1):119. doi:10.1186/s12882-021-02319-9. 33827478 PMC8028152

[10] Dsouza B, Prabhu R, Unnikrishnan B, et al. Effect of educational intervention on knowledge and level of adherence among hemodialysis patients: a randomized controlled trial. Glob Health Epidemiol Genom. 2023;2023:4295613. doi:10.1155/2023/4295613. 37033597 PMC10081894

[11] Griva K, Nandakumar M, Ng JH, et al. Hemodialysis Self-management Intervention Randomized Trial (HED-SMART): a practical low-intensity intervention to improve adherence and clinical markers in patients receiving hemodialysis. Am J Kidney Dis. 2018;71(3):371–381. doi:10.1053/j.ajkd.2017.09.014. 29198641

[12] Xu F, Zhuang B, Wang Z, et al. Knowledge, attitude, and practice of patients receiving maintenance hemodialysis regarding hemodialysis and its complications: a single-center, cross-sectional study in Nanjing. BMC Nephrol. 2023;24(1):275. doi:10.1186/s12882-023-03320-0. 37730535 PMC10510168

[13] Torabikhah M, Farsi Z, Sajadi SA. Comparing the effects of mHealth app use and face-to-face training on the clinical and laboratory parameters of dietary and fluid intake adherence in hemodialysis patients: a randomized clinical trial. BMC Nephrol. 2023;24(1):194. doi:10.1186/s12882-023-03246-7. 37386428 PMC10308810

[14] Deng Y, Li N, Wu Y, et al. Global, regional, and national burden of diabetes-related chronic kidney disease from 1990 to 2019. Front Endocrinol (Lausanne). 2021;12:672350. doi:10.3389/fendo.2021.672350. 34276558 PMC8281340

[15] Mirzaei-Alavijeh M, Hamzeh B, Omrani H, et al. Determinants of medication adherence in hemodialysis patients: a cross-sectional study based on capability-opportunity-motivation and behavior model. BMC Nephrol. 2023;24(1):174. doi:10.1186/s12882-023-03231-0. 37316774 PMC10266875

[16] Wheeler DC, Stefánsson BV, Jongs N, et al. Effects of dapagliflozin on major adverse kidney and cardiovascular events in patients with diabetic and non-diabetic chronic kidney disease: a prespecified analysis from the DAPA-CKD trial. Lancet Diabetes Endocrinol. 2021;9(1):22–31. doi:10.1016/S2213-8587(20)30369-7. 33338413

[17] Komiya S, Katsumata M, Ozawa M, et al. Efficacy of tolvaptan on advanced chronic kidney disease with heart failure: a randomized controlled trial. Clin Exp Nephrol. 2022;26(9):851–858. doi:10.1007/s10157-022-02224-x. 35471469 PMC9385808

[18] Hamidi M, Roshangar F, Khosroshahi HT, et al. Comparison of the effect of linear and step-wise sodium and ultrafiltration profiling on dialysis adequacy in patients undergoing hemodialysis. Saudi J Kidney Dis Transpl. 2020;31(1):44–52. doi:10.4103/1319-2442.279960. 32129196

[19] Chan K, Wong F, Tam SL, et al. Effectiveness of a brief hope intervention for chronic kidney disease patients on the decisional conflict and quality of life: a pilot randomized controlled trial. BMC Nephrol. 2022;23(1):209. doi:10.1186/s12882-022-02830-7. 35701732 PMC9195369

[20] Valsaraj BP, Bhat SM, Prabhu R, et al. Follow-up study on the effect of cognitive behaviour therapy on haemodialysis adherence: a randomised controlled trial. Sultan Qaboos Univ Med J. 2021;21(1):e58–e65. doi:10.18295/squmj.2021.21.01.008. 33777424 PMC7968912

[21] Kwan BC, Szeto CC, Chow KM, et al. Bioimpedance spectroscopy for the detection of fluid overload in Chinese peritoneal dialysis patients. Perit Dial Int. 2014;34(4):409–416. doi:10.3747/pdi.2013.00066. 24385329 PMC4079487

